# Upper Respiratory Tract Colonization by Gram-Negative Rods in Patients with Chronic Lymphocytic Leukemia: Analysis of Risk Factors

**DOI:** 10.1100/2012/617218

**Published:** 2012-04-19

**Authors:** Izabela Korona-Glowniak, Ewelina Grywalska, Beata Chudzik, Agnieszka Bojarska-Junak, Anna Malm, Jacek Rolinski

**Affiliations:** ^1^Department of Pharmaceutical Microbiology, Medical University of Lublin, 20-093 Lublin, Poland; ^2^Department of Clinical Immunology, Medical University of Lublin, 20-093 Lublin, Poland

## Abstract

The aim of the study was to assess the frequency and predisposing factors of colonization of upper respiratory tract by Gram-negative rods (GNRs) in chronic lymphocytic leukemia (CLL) patients. Antimicrobial susceptibility of the isolated strains was determined. A significantly higher frequency of GNR colonization in CLL patients was observed (36.7%) in comparison to healthy volunteers (8.3%). GNR isolates mainly belonged to the Enterobacteriaceae family. Three isolates of GNR demonstrating presence of AmpC **β**-lactamases and one ESBL-producing strain were obtained from CLL patients. GNR colonization rate was higher among CLL patients with lower level of IgG in serum (*P* = 0.017), with higher number of neutrophils (*P* = 0.039) or higher number of lymphocytes in serum (*P* = 0.053). The longer the time elapsed since diagnosis, the higher the frequency of GNR colonization observed. Multivariate analysis showed importance of the Rai stage, number, and type of infections as independent predictors of GNR colonization in CLL patients.

## 1. Introduction

Infectious complications are still one of the major causes of morbidity and mortality in patients with chronic lymphocytic leukemia (CLL). Infections affect mainly the respiratory tract, skin, or urinary tract. The most common respiratory infections are acute and chronic sinusitis, otitis media, and pneumonia. In the past pneumonia was caused mainly by *Streptococcus pneumoniae*, but with current chemotherapeutic regimens, the spectrum of pathogens includes Gram-negative rods (GNR), *Nocardia* species, *Legionella* species, and *Pneumocystis carinii* [1]. An increase in infections caused by GNR, particularly bacteremia and pneumonia, may reflect more advanced disease and profound myelosuppression in these patients [2].

The epithelial surfaces of the upper respiratory tract are continuously exposed to a wide variety of commensal and potentially pathogenic microorganisms, but the density and composition of colonization need to be controlled by the host [3]. In addition to acting as a physical barrier, epithelial cells respond to specific microbial products with the generation of signals, such as cytokines, that trigger inflammation. Colonization of the upper respiratory tract by pathogens is often the first step in a multifactorial process leading to disease. About 80% of CLL patients will sustain infectious complications during their disease, and 50–60% of patients will die due to infection [4]. The major risk factors for infection in these patients are immune defects that are inherent to the primary disease process and therapy-related immunosuppression. Refractory CLL patients subjected to allogenic hematopoietic cell transplantation (allo-HCT) also have a particularly high incidence of infections compared to allo-HCT recipients with other lymphoid malignancies [5, 6] Disease- and therapy-related immune defects include hypogammaglobulinemia, as well as perturbations in cell-mediated immunity, complement activity, and neutrophil function [4, 7].

The aim of this study was to assess the frequency and predisposing factors of colonization of upper respiratory tract by GNR in previously untreated CLL patients. Antimicrobial susceptibility of the isolated strains was determined. To the best of our knowledge, the present study is the first report analyzing this issue.

## 2. Material and Methods

### 2.1. Patients

This prospective study included 30 previously untreated patients with CLL and 24 healthy volunteers attending the Department of Clinical Immunology, Medical University of Lublin, from March to August 2011. Information regarding baseline characteristics, stage according to the Rai staging system [8], hematological tests results, and number and types of infections per year (upper or lower respiratory tract infections, skin infections) were determined for CLL patients to analyze risk factors of upper respiratory colonization by GNR in this group (Table 1).

### 2.2. Microbiological Procedures

Throat and nasal specimens were taken using sterile alginate-tipped swabs from each of the patients during control visit in clinical health care settings. Swabs were placed directly into Stuart's trans-medium, and samples were delivered to the laboratory, where specimens were streaked onto nonselective medium (blood agar) and selective medium (MacConkey agar). Plates were incubated for 24–48 h at 35°C under aerobic conditions. Presence of GNR in sample from nostrils and/or throat was called colonization. The identification of isolates was determined using Api 20E or Api 20NE as appropriate. Antimicrobial susceptibility of the isolates was tested by the disc diffusion method according to the European Committee on Antimicrobial Susceptibility Testing recommendations (EUCAST 2011). ESBL production screening was detected by double-disk synergy test [9].

### 2.3. Flow Cytometric Analysis

Peripheral blood (PB) specimens were obtained from 30 CLL patients and was taken to 10 mL tubes with an EDTA anticoagulant. All PB samples were stained for ZAP-70 protein expression and CD38 antigen expression, as described previously [10]. A cut-off point for ZAP-70 positivity was ≥20%, for CD38 positivity was ≥30% [11] and for CD25 positivity in leukemic cells was ≥20%. Three-color immunofluorescence analyses were performed using a FACS Calibur flow cytometer (Becton Dickinson) equipped with 488 nm argon laser. A minimum of 10,000 events was acquired and analyzed using CellQuest Software. Mean fluorescence intensity (MFI) and the percentage of cells expressing surface markers were analyzed. The cells were phenotypically characterised by incubation (20 min in the dark at a room temperature) with combination of relevant fluorescein-isothiocyanate- (FITC-), phycoerythrin- (PE-), and CyChrome-labeled monoclonal antibodies (mAbs). Immunofluorescence studies were performed using a combination of the following mAbs: CD3 FITC/CD19 PE, CD8 FITC/CD4 PE, CD25 CyChrome, and CD69 CyChrome. An acquisition gate was established based on FSC and SSC that excluded dead cells and debris. Isotype-matched antibody was used to verify staining specificity and as a guide for setting markers used to delineate positive and negative populations.

### 2.4. Statistical Analysis

Data processing and analysis were performed using Statistica 7.0 PL software, StatSoft Inc. The results are expressed as percentage and mean ± SD. Continuous variables were compared using a nonparametric test (*U* Mann-Whitney test) or parametric Student *t*-test and categorical variables by Chi-square or the Fisher exact test, as appropriate. Relative risks (RRs) and their 95% confidence intervals (CIs) were calculated. Statistical significance was set at *P* < 0.05. Logistic regression was performed to evaluate the risk factors associated with GNR colonization. Potential predictor variables for model entry were identified using univariate analysis. Regression models were controlled for the effects of confounding variables. Results of the logistic regression analysis are reported as adjusted odds ratio (OR) with 95% CI.

## 3. Results

A total of 108 samples obtained from upper respiratory tract of 54 persons (30 patients with CLL and 24 healthy volunteers) were bacteriologically examined. Colonization by GNR was observed in 24.1% of the studied persons. A significantly higher frequency of GNR colonization in CLL patients (36.7%) was observed in comparison to healthy volunteers (8.3%). This difference was statistically significant (*P* = 0.02; RR 4.4; 95%CI 1.1–18.0).

The overall 16 isolates of GNR (from 3 patients, 2 different species of GNR were isolated) were cultured: 8 (50%) isolates were obtained from throat, 6 (37.5%) from nostrils, and 2 (12.5%) isolates colonized both throat and nostrils. Species of GNR isolates are shown in Table 2. GNR isolates mainly belonged to the Enterobacteriaceae family, and only 1 isolate, *Pseudomonas aeruginosa*, belonged to nonfermentative Gram-negative rod—Pseudomonadaceae.

ESBL-producing isolate, *Proteus vulgaris*, was obtained from one CLL patient. Four isolates, *Citrobacter koseri, Serratia marcescens*, and two isolates of *Hafnia alvei*, demonstrated the presence of AmpC *β*-lactamases (Table 2), three of them were obtained from CLL patients.

The selected factors were tested for their association with GRN colonization in CLL patients. At the first, in univariate analysis, the differences in hematological parameters of colonized by GNRs and noncolonized patients turn out to be statistically significant (Table 3). Colonization rate was higher among CLL patients with lower level of IgG in serum (*P* = 0.017) and patients with higher number of neutrophils (*P* = 0.039) or those who had higher number of lymphocytes in serum (*P* = 0.053). It was shown that the longer the time elapsed since diagnosis in CLL patients, the higher the frequency of GNR colonization observed (*P* = 0.025). Multivariate analysis showed importance of the Rai stage, number of infections per year, and type of infections as independent predictors of upper respiratory GNR colonization in CLL patients (Table 4).

## 4. Discussion

Colonization of the respiratory tract by Gram-negative bacteria is a frequent cause of infection, mainly pneumonia. In healthy individuals, the upper respiratory tract is not usually colonized by GNR [12]. Prevalence of GNR colonization increases with the presence of several serious illnesses. Johanson et al. [13] reported the direct relation of oropharyngeal GNR colonization and the severity of underlying illness of the population studied. Colonization rates of three studied groups, healthy person, moderately ill patients, and patients classified as “moribund”, were 2%, 16%, and 57%, respectively. In our study, about 8% of healthy volunteers and about 37% of CLL patients were colonized by GNR. This data are similar to those obtained by Los et al. [14] in patients with lung cancer. Also similar results were shown by Ortqvist et al. [15] who conducted study on patients with secondary infection during hospital treatment. It was shown that 35% patients were colonized by GNR during hospitalization, and this colonization was a negative prognostic factor, associated with higher mortality, doubled length of hospital stay, and slower recovery. During hospitalization, patient's microflora is constantly changing depending on the environmental condition and applied treatment. Fermentative and nonfermentative GNR which are often present in hospital microflora colonize patients successfully. Patients tested in our study were untreated and not hospitalized of any cause, so frequency of GNR colonization seems to be closely related only to the patient status. Niederman [12] suggested that colonization of the oropharynx by Gram-negative bacteria is a marker for critically ill patients who have multiple deficiencies in the host defense system of their respiratory tract, and these patients are also likely to have other impairments in the cellular and humoral response to bacterial invasion.

There is no data related to impact and significance of colonization in upper respiratory tract CLL patients by GNR to develop infection during the course of the disease. Patients with CLL have defected humoral and cellular immunity, damages in the complement system, and variable neutropenia that contribute to the high rate of infections. Hypogammaglobulinemia and advanced disease are major predisposing factors described elsewhere [1, 4, 16]. In patients having early-stage untreated CLL, risk of infections may be related mainly to hypogammaglobulinemia [2]. Hypogammaglobulinemia is one of the two routinely measured immune defects associated with CLL (the other one is neutropenia), which correlates with disease duration and stage. Its association with infection in CLL patients is well known [2, 4, 17]. In our study, lower level of IgG in serum and higher Rai stage of CLL were related to higher frequency of colonization by GNR. Additionally, statistically significant predictor for GNR colonization was longer period from diagnosis of CLL in studied patients.

A significant correlation between low IgA level and the risk of infections was demonstrated in some reports [18, 19]. In our patients with CLL, lower level of IgA in the group of GNR colonized patients in comparison to noncolonized group was observed, but this difference was not statistically significant.

Despite known defects in cell-mediated immunity and complement activity, no correlation has been proved between these defects and the occurrence of infectious complication or bacterial colonization. In our study, no relation between GNR colonization and particular cell subsets was observed. The T-cell abnormalities found in CLL patients are thought to increase the risk of infection and hamper immune recognition and elimination of leukemic cells. Bonyhadi et al. [20] suggested that in vitro engagement of CD3 and CD28 corrects T-cell defects in CLL. Researchers found that peripheral blood mononuclear cells incubated with anti-CD3 and anti-CD28 monoclonal antibodies conjugated to superparamagnetic beads for 12–14 days caused a 1400-fold increase in T-cell numbers. Activated T cells expressed high levels of CD25, CD54, CD137, and CD154 and produced IFN-*γ*, TNF-*α*, and GM-CSF. The mean T-cell composition of cultures increased from around 6% to >90%. Aforementioned authors initiated also a clinical trial to test the potential therapeutic effects of T cells activated using this approach in patients with CLL. Thus, infection rates may be diminished; however, this method will probably not affect GNR colonization of nasopharynx. In patients with *Shigella *infection, Islam et al. [21] found evidence for sequential T-cell activation, as shown by increased proportions of CD25 and CD4+ cells, HLA-DR and CD38 on CD8+ cells, and CD54 on CD4+ and CD8+ cells. Neither the proportion of CD3 T cells in peripheral blood mononuclear cells nor the CD3 MFI was significantly different in the group of infected individuals than it was in controls, and our observations are similar to theirs. In the research of Stuller et al. [22], it was demonstrated that CD25(+) cells induce anergy in CD25(−) cells in response to *Helicobacter pylori *infection but are not required to maintain hyporesponsiveness. In addition, CD25(+) cells are able to suppress previously activated CD25(−) cells when responding to this bacteria challenge in vivo. The lack of GNR colonization influence on activation markers in CLL patients may be due to disease-dependent cell dysfunction or bacteria-induced immunosuppression.

We performed also an analysis of principal CLL prognostic factors in order to determine if the percentage of ZAP-70+ and CD38+ cells differ in colonized and not colonized patients. High levels of CD38 and ZAP-70 expression [11, 23] and unmutated immunoglobulin (Ig)VH genes [11, 24] are associated with aggressive disease. Francis et al. [25] found that older age, clinical stage of the disease, unmutated IgVH gene status, and positive CD38 status were associated with a significantly shorter time to first infection. Authors also maintained that patient age, disease stage, CD38 expression, and IgVH mutation status had a significant impact on infection-related mortality. Our study revealed that the Rai stage correspond with potentially pathogenic bacteria colonization of upper respiratory tract, so the patients present higher infection risk. Higher number of lymphocytes was observed in our study as the predictor of GNR colonization in CLL patients and the amount of these cells, especially lymphocyte doubling time, are predictors of a shorter treatment-free survival [26]. Therefore, we propose to administrate to the colonized individuals prophylaxis with the use of appropriate antibiotics.

Our data indicate that patients colonized by GNR had significantly higher absolute neutrophil count than noncolonized ones. Quantitative and qualitative defects of neutrophils are observed in CLL patients, but their total number is normal or slightly decreased in untreated patients and may decline further with disease progression, due to marrow infiltration and use of myelosuppressive therapy. Although neutrophil function seems to be normal in CLL patients, enzyme deficiencies in the neutrophils of untreated patients leading to inhibition of neutrophil phagocytic and bactericidal activity were demonstrated [4].

A better determinant of association between CLL and upper respiratory bacterial colonization should be an assessment of the mucosal immune defects. We did not analyse levels of mucosal (salivary) IgA, IgG, and IgM in tested CLL patients, but in preliminary report of other authors no associations were found between salivary immunoglobulin levels and infections [17].

As expected, in our study infections were frequent in CLL patients, but we were not able to find a correlation between specific colonized strain and specific infection. However, higher number of infections and type of infection were shown as independent predictors of GNR colonization in CLL patients. (Upper respiratory GNR colonization is not the synonymous of infection but indicates the higher risk of its development in immunodeficient patients especially.)

Changing spectrum of bacterial pathogens in infected patients with neutropenia and cancer over the past 3 decades was observed. In most medical centers, Gram-negative bacteria, for example, *Escherichia coli, Pseudomonas aeruginosa*, and *Klebsiella *spp., were responsible for 60%–70% of infections. Recently, a clear shift in these infecting organisms was documented, and mainly Gram-positive cocci were responsible for bacteremia in patients with neutropenia. Zinner [27] suggested that some of the factors believed responsible for this shift include oral mucositis as a result of increasingly potent chemotherapeutic regiments, profound and prolonged neutropenia, and factors connected to hospitalization and therapy. Patients tested in our studied were untreated due to CLL so none of them was exposed to mentioned factors and high rate of GNR colonization was observed.

In our study, 4 isolates of GNRs demonstrating presence of extended-spectrum *β*-lactamases were obtained from CLL patients—ESBL-producing strain, and three AmpC-presented strains. AmpC *β*-lactamases are clinically important cephalosporinases encoded on the chromosome in many Enterobacteriaceae and a few other organisms, and they mediated resistance to multiple agents, making the selection of an effective antibiotic therapy difficult. *β*-lactam/*β*-lactamase inhibitor combinations and most penicillins and cephalosporins should be avoided because of in vitro resistance and the potential for AmpC induction or selection of high-enzyme-level mutants [28]. ESBLs are enzymes that mediate resistance to extended-spectrum (third generation) cephalosporins of third generation (e.g., ceftazidime, cefotaxime, and ceftriaxone) and monobactams (e.g., aztreonam) but do not affect cephamycins (e.g., cefoxitin and cefotetan) or carbapenems (e.g., meropenem or imipenem) [29]. ESBL-producing organisms pose a major problem for clinical therapeutics. The incidence of *β*-lactamases-producing strains among clinical isolates has been steadily increasing over the past decade resulting in limitation of therapeutic options. Initially restricted to hospital acquired infections, they have also been isolated from infections in outpatients. Major outbreaks involving ESBL strains have been reported all over the world, thus making them emerging pathogens.

## 5. Conclusion

CLL patients are not a homogeneous population in respect of infectious morbidity and mortality. Therefore, awareness of risk factors predisposing to pathogens colonization, for example, upper respiratory GNR colonization, allows to identify group of patients which should be considered for Ig or antibiotic prophylaxis. Moreover, knowledge about antibiotic resistance of the colonizing pathogens is important to propose not only optimal antibiotic prophylaxis scheme but also empiric and targeted therapy with greater likelihood of clinical success.

## Figures and Tables

**Table 1:** Selected demographic, clinical, and laboratory parameters of the CLL patients.

Parameter	No. of patients (%)	Mean ± SD	Range
Age		60.47 ± 8.3	43–80
Gender:			
Males	12 (40)		
Females	18 (60)		
The Rai stage:			
0	8 (26.7)		
1	8 (26.7)		
2	11 (36.7)		
3	3 (10.0)		
Period from diagnosis (years)		5.0 ± 3.3	0–11
No. of infections per year		4.4 ± 3.1	1–11
Type of infection:			
URTIs	12 (40.0)		
LRTIs	5 (16.7)		
URTs+LRTIs	6 (20.0)		
LTRIs+skin infections	1 (3.3)		
URTIs+LRTIs+skin infections	2 (6.7)		
Lymphocytes (%)		72.1 ± 11.8	48.3–89.3
Lymphocytes (10^3^/mm^3^)		27.2 ± 23.3	5.3–90.9
Neutrophils (%)		22.1 ± 17.4	3.5–91.0
Neutrophils (10^3^/mm^3^)		6.1 ± 4.9	1.2–20.5
IgA (mg/dL)		153.0 ± 103.2	3–380
IgG (mg/dL)		774.3 ± 303.1	320–1616
IgM (mg/dL)		64.3 ± 52.8	4–220
CD3^+^ cells (%)		13.8 ± 11.9	1.2–56.2
CD19^+^ cells (%)		80.6 ± 13.5	35.2–96.8
CD5^+^CD19^+^ cells (%)		74.6 ± 20.2	45–96.7
CD19^+^ZAP70^+^ cells (%)		16.2 ± 13.1	1.7–45.3
Negative	21 (75.0)		
Positive	7 (25.0)		
CD19^+^CD38^+^ cells (%)		17.7 ± 18.7	0.3–56.7
Negative	20 (71.4)		
Positive	8 (28.6)		
ZAP70 expression in relation to CD38 expression on B lymphocytes			
ZAP70 (−), CD38 (−)	18 (64.3)		
ZAP70 (+), CD38 (+)	5 (17.9)		
ZAP70 (+), CD38 (−)	2 (7.1)		
ZAP70 (−), CD38 (+)	3 (10.7)		
CD19^+^CD25^+^ cells (%)		39.0 ± 25.5	1.9–89.8
Negative	7 (25.0)		
Positive	21(75.0)		
CD19^+^CD69^+^ cells (%)		23.4 ± 18.3	0.4–60.8
CD3^+^CD25^+^ cells (%)		20.5 ± 12.5	2.4–52.8
CD3^+^CD69^+^ cells (%)		6.2 ± 7.3	0.5–29.3
CD3^+^CD4^+^cells (%)		7.5 ± 6.4	0.8–33.1
CD3^+^CD8^+^cells (%)		7.0 ± 6.1	0.5–24.2
CD4^+^ to CD8^+^ cells ratio		1.4 ± 0.7	0.3–3.2
CD4^+^CD8^+^cells (%)		0.5 ± 0.7	0.03–3.6

URTIs: upper respiratory tract infections, LRTIs: lower respiratory tract infections.

**Table 2:** Gram-negative rods isolated from upper respiratory tract from examined patients and their resistance patterns.

Species	No. of isolates	Resistance pattern (no. of isolates)
*Escherichia coli *	4	S (4)
*Klebsiella pneumoniae *subsp.* pneumoniae *	2	S (1); Am (1)
* Klebsiella oxytoca *	2	Am Pip Tic Tob (1); Am Pip Tic (1)
*Serratia marcescens *	1	Am Amc Pip Tzp Tic Tim Cxm Ctx Caz Te
*Proteus vulgaris *	1	Am Amc Pip Tzp Tic Tim Cxm Ctx Caz Fep Atm Ipm Te Sxt
*Proteus mirabilis *	1	Cxm
*Citrobacter koseri *	1	Am Amc Pip Tzp Tic Tim Cxm Ctx Caz
*Hafnia alvei *	1	Am Amc Pip Tzp Tic Tim Cxm Ctx Caz
*Pseudomonas aeruginosa*	1	Amc Te Sxt

Am, ampicillin; Amc, amoxycillin/clavulanate acid; Pip, piperacillin; Tzp, piperacillin/tazobactam; Tic, ticarcillin; Tim, ticarcillin/clavulanate acid; Cxm, cefuroxime; Ctx, cefotaxime; Caz, ceftazidime; Fep, cefepime; Atm, aztreonam; Ipm, imipenem; Te, tetracycline; Sxt, trimetoprim/sulfamethoxazole.

**Table 3:** The association between Gram-negative rods colonization and selected demographic, clinical or laboratory parameters in CLL patients.

Predictors	No. of patients or mean ± SD		*P*; OR (95%CI)
	GNR colonized (*n* = 11)	Noncolonized (*n* = 19)	
Age	58.2 ± 6.3	59.9 ± 9.8	0.73
Males	2 (18.2)	10 (52.6)	0.12; 1.7 (1.0–3.0)
Rai stage:			0.03*
0	1 (9.1)	7 (36.8)	
1	1 (9.1)	7 (36.8)	
2	7 (63.6)	4 (21.1)	
3	2 (18.2)	1 (5.3)	
Period from diagnosis (years)	6.8 ± 2.1	3.9 ± 3.3	0.025*
No. of infections per year	3.9 ± 1.9	3.6 ± 2.2	0.26
Types of infections			0.22
Lymphocytes (%)	71.1 ± 11.9	71.6 ± 11.6	0.83
Lymphocytes (10^3^/mm^3^)	39.2 ± 23.5	16.4 ± 9.0	0.053
Neutrophils (%)	20.0 ± 11.7	19.9 ± 11.3	0.85
Neutrophils (10^3^/mm^3^)	7.5 ± 4.0	5.3 ± 5.3	0.039*
IgA (mg/dL)	90.3 ± 45.3	146.4 ± 100.2	0.21
IgG (mg/dL)	563.3 ± 217.5	940.0 ± 307.5	0.017*
IgM (mg/dL)	31.5 ± 14.8	62.1 ± 50.6	0.95
CD3^+^ cells (%)	13.4 ± 5.3	11.7 ± 7.9	0.86
CD19^+^ cells (%)	80.5 ± 7.4	84.1 ± 8.9	0.60
CD5^+^CD19^+^ cells (%)	18.9 ± 8.8	72.5 ± 24.6	0.55
CD19^+^ZAP70^+^ cells (%)	14.5 ± 14.5	18.3 ± 14.1	0.77
Positive/negative	3/4 (42.9)	7/14 (33.3)	0.67; 1.5 (0.3–1.6)
CD19^+^CD38^+^ cells (%)	21.3 ± 17.1	18.2 ± 19.1	0.38
Positive/negative	4/4 (50.0)	6/14 (30.0)	0.40; 2.3 (0.4–12.6)
CD19^+^CD25^+^ cells (%)	26.2 ± 23.4	44.2 ± 27.1	0.14
Positive/negative	8/13 (38.1)	2/5 (28.6)	1.0; 1.5 (0.2–9.9)
CD19^+^CD69^+^ cells (%)	16.0 ± 13.1	21.6 ± 20.7	0.88
CD3^+^CD25^+^ cells (%)	18.9 ± 8.8	18.5 ± 8.3	0.72
CD3^+^CD69^+^ cells (%)	2.6 ± 1.3	7.6 ± 7.9	0.55
CD3^+^CD4^+^cells (%)	8.3 ± 3.2	6.1 ± 3.3	0.19
CD3^+^CD8^+^cells (%)	7.3 ± 4.1	7.3 ± 6.1	0.98
CD4^+^-to-CD8^+^ cells ratio	1.3 ± 0.7	1.3 ± 0.8	0.95
CD4^+^CD8^+^cells (%)	0.4 ± 0.1	0.4 ± 0.4	1.0

__*P < 0.05.

**Table 4:** Logistic regression analysis of factors predictive of GNR colonization in CLL patients.

Predictors	OR (95%CI)	*P*
Rai stage	8.1 (1.4–47.6)	0.014
No. of infections per year	4.2 (1.1–15.8)	0.027
Types of infections	0.06 (0.005–0.7)	0.018
